# Before progressing from “exomes” to “genomes”… don’t forget splicing variants

**DOI:** 10.1038/s41431-018-0214-3

**Published:** 2018-07-12

**Authors:** Samiha S. Shaikh, Michael S. Nahorski, Harjeet Rai, C. Geoffrey Woods

**Affiliations:** 1 Department of Medical Genetics, Cambridge Institute for Medical Research, Hills Road, Cambridge, CB2 0XY United Kingdom; 2 East Anglian Medical Genetics Service, Genetics Laboratories, Addenbrooke’s Treatment Centre, Cambridge University Hospitals, Hills Road, Cambridge, CB2 0QQ United Kingdom

Splicing variants are less commonly reported than other variant types [1–3]. However, despite most being functional nulls, previous reports suggested that they are under-recognized [2]. Our recent experience of investigating a cohort of 38 individuals with a severe, genetically heterogeneous Mendelian phenotype shows that this continues to be a problem; three variants that affected splicing were initially “missed” because they were not detected by current splice site detection algorithms. Our concern is that splicing variants will continue to be overlooked in clinical laboratory settings because the quantity of data generated per person by “exomes” and “genomes” necessitates the use of splice site detection programs. Our cases highlight significant deficiencies in current standard programs, where variants at the U2 canonical AG (acceptor) and GT (donor) splice sites are reliably detected, but variants at other positions with more loosely defined consensus sequences, or U12 splice sites, are rarely detected [4].

We analyzed 38 sequentially ascertained samples from individuals who were born unable to feel pain within an UK NHS genetic service. We initially found 28 of the 38 cases had bi-allelic variants that affected function in *SCN9A* (17), *NTRK1* (13) and *NGF* (1); all causing autosomal recessive painless disorders. Given the specific phenotype and limited genotypes, we hand-curated the remaining cases. In three unrelated index individuals rare variants within intronic regions were present on sequencing (ExAC frequencies of 2/113990, 0/61864, and 2/120742). Splice site prediction program analysis of each variant was performed using Alamut (http://www.interactive-biosoftware.com/alamut-visual/), which incorporates five different programs all of which have been developed for U2 splice sites rather than U12 splice sites (see Table 1). Whilst the three variants were not flagged, we considered they could alter splice site function due to their proximity to known splice sites, and assessed each by a minigene splicing assay [5], as transcriptomic sequencing was not possible (see Supplement for methodology).

**Table 1:** Summary of the results of splice prediction programs for detecting the wild-type splice sites, and the effects of variants on each

Was the variant predicted to alter the splice site by	*NTRK1* c.575–19 G > A Exon 6 acceptor site	*NTRK1* c.717 + 4 A > T Exon 6 donor site	*SCN9A* c.377 + 5 C > T Exon 3 donor site
MaxEntScan (1–16)	Yes: 5 to 3.7	Yes: 7.2 to 1.7	No: ND
SpliceSiteFinder-like (0–100)	No: 75 to 75	No: 81 to 71	No: ND
Human Splicing Finder (0–100)	No: 82.5 to 82.5	No: 91 to 82	No: ND
GeneSplicer (0–15)	No: 2.5 to 2.5	Yes: 9.2 to 2.5	No: ND
NNSplice (0–1)	No: 0.9 to 0.9	Yes: 0.7 to 0	No: ND
Spliceman (0–100)	Yes: 75	Yes: 55	Yes: 82

The Alamut program, which incorporates MaxEntScan, SpliceSiteFinder-like, Human Splicing Finder, GeneSplicer and NNSplice, was used as the primary assessment tool for the intronic variants. For each prediction program in Alamut we have indicated the scale range in brackets and have stated whether the variants were predicted to be deleterious in bold if the change in variation was greater than 15% (yes/no) and given the score of the wild-type splice site and the effect of the variant below the prediction. At least three of the five programs had to strongly predict an effect on splicing at the canonical splice site in order to be considered as disease-causing by the clinical laboratory.

The *NTRK1* c.575–19 G > A variant was only predicted to have deleterious effects on the wildtype acceptor site by one program, MaxEntScan. The *NTRK1* c.717 + 4 A > T variant had deleterious predictions in three programs but as the +4 position was known to be the least conserved it was not flagged as being likely to affect function. The *SCN9A* exon 3 splice site was not detected as a splice site by any of the five programs in Alamut, and hence the effect of the variant could not be determined. This was unsurprising as the intron 3 of *SCN9A* is a U12 intron, for which none of the Alamut programs were designed. However, the more recently written splice prediction program Spliceman did predicted the splice site and the deleterious effect of the variant. Spliceman reports variants as a percentage; the higher the percentile rank, the more likely it is the variation will disrupt splicing.

ND: wildtype splice was not detected by the program

Each variant was proven to alter splicing by comparing the results to those of normal wild-type splicing (see Fig. 1). The first case had a hereditary sensory and autonomic neuropathy type 4 (HSAN4) phenotype but sequencing analysis had detected no variants, however we noted a homozygous *NTRK1* variant, c.575–19 G > A (reference sequence NM_002529.3; exons are numbered as in the reference sequence NG_007493.1) [6]. Bioinformatics analysis suggested that this potentially created a new AG splice acceptor site, which was predicted to be superior to the existing site; this was the case, with the introduction of a 17 bp frame-shifting insertion into the *NTRK1* mRNA (Fig. 1b.ii). The second case also had a HSAN4 phenotype but sequencing had revealed only one *NTRK1* heterozygous variant proven to affect function c.1550 G > A^6^. On sequence inspection we noted a heterozygous splice donor site variant c.717 + 4 A > T. Although usually + 4 can be any base, in *NTRK1* this + 4 position is invariant [7]. The minigene assay showed that the variant resulted in complete loss of *NTRK1* exon 6 in the mature transcript (Fig. 1b.iii). The third case had a phenotype consistent with congenital insensitivity to pain (CIP)—a lack of pain and smell perception with normal intelligence. Only a single heterozygous variant in *SCN9A* was detected, c.2686 C > T (reference sequence NM_002977.3; exons are numbered as in the reference sequence NG_012798.1). On inspection of the sequencing data we noted a heterozygous variant c.377 + 5 C > T in *SCN9A* occurring in a U12 splice site. The U12 donor site sequence is RTATCCTT where +5 C is invariant [8], in contrast to the more ubiquitous U2 splice site where +5 can vary [7]. The variant caused complete loss of exon 3 and aberrant splicing into a cryptic U2 acceptor site resulting in a +1 frame shift (Fig. 1c.ii). All three variants were predicted to lead to nonsense-mediated decay and hence be nulls. Early nonsense and frame shift variants have been identified in other cases of HSAN4 and CIP patients and hence are likely to explain the disease in our patients [9].

**Fig. 1:**
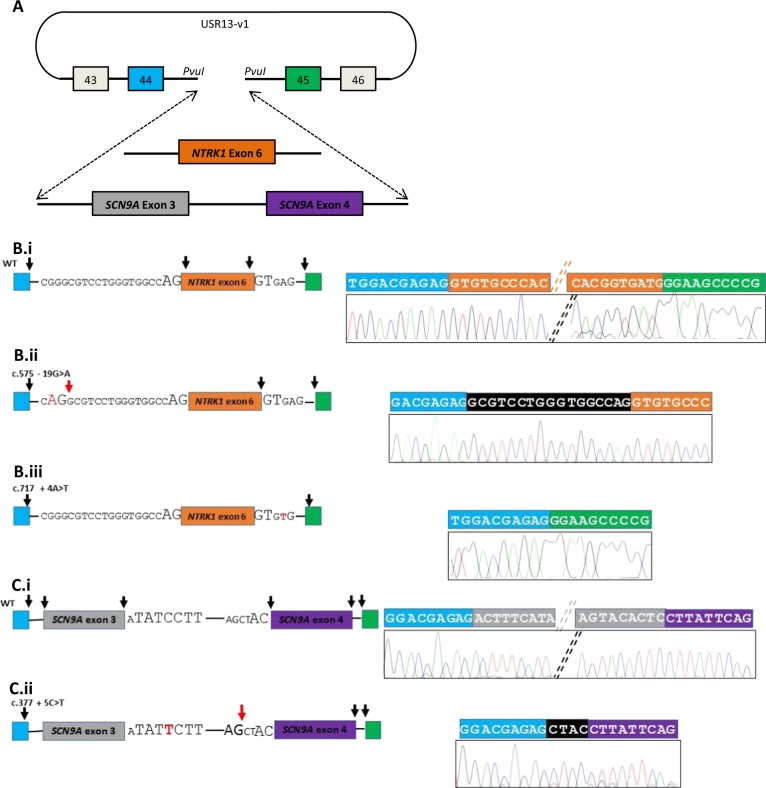
Summary of minigene assay and sequencing results demonstrating the functional consequences of three “missed” splicing variants. The USR13-v1 vector, used for minigene assays, (**a**) contains *COL2A1* exons 43–46 and intervening introns. Between exon 44 and 45, a multiple cloning site is present to allow cloning of the test region. *NTRK1* exon 6 and flanking intronic regions were introduced into the vector. *SCN9A* exon 3, intron 3–4 and exon 4, as well as flanking intronic regions were introduced into the vector. Exons are shown as colored boxes; blue and green for the minigene exons (exon 44 and 45 of *COL2A1*), orange for *NTRK1* exon 6, and grey and purple for *SCN9A* exons 3 and 4. Mini-gene constructs of wild-type and mutant *NTRK1* exon 6 (**b**) and *SCN9A* exon 3 and 4 (**c**), with their flanking intronic regions, were expressed in HeLa cells. PCR was performed on cDNA converted from extracted mRNA and sequenced. For each reaction the left panel is a schematic of the splicing event and the right panel an annotated chromatogram of sequence from the minigene PCR reaction. The splice acceptor and donor site nucleotides are shown surrounding the exons studied, with the invariant nucleotides enlarged. The variants investigated are shown in red. Black arrows indicate normal splicing. For each variant, a loss of a black arrow indicated that splicing at that site failed to occur, and red arrows indicate new splice sites formed because of the variants. **b**.i details the normal splicing of *NTRK1* exon 6. **b**.ii shows the effect of c.575–19 G > A producing a novel splice acceptor site 19 bp from the start of exon 6 - resulting in the addition of 17 nucleotides into the mRNA, a frameshift and a premature stop codon. **b**.iii shows the effect of **c**.717 + 4 A > T, a splice donor variant at +4 bp: the whole of exon 6 is missing from the final transcript resulting in a frame-shift in the reading frame of the mRNA and a premature stop codon. **c**.i details the normal splicing event for *SCN9A* exon 3 and 4, where intron 3 has U12 splicing. **c**.ii shows the effect of c.377 + 5 C > T in the +5 site in the U12 donor site sequence of *SCN9A* intron 3. This lead to a complete failure of splicing of exon 3, and the use of a cryptic U2 splice donor site prior to the U12 splice acceptor site of exon 4 resulting in 4 bp of intron 3 being added to exon 4. This resulted in a + 1 frame shift, a stop codon at the 21st codon of exon 4, and nonsense-mediated decay

In our cohort, a tenth of cases harbored missed variants that affected splicing. Their detection was considerably aided by the clear phenotype and the limited number of genes that required analysis. Had the phenotype been more variable, or did not resemble a single-gene disorder, or the number of potentially causative genes greater, it is possible that these variants would have gone undetected. These cases also illustrate the need to consider seeking a second variant in autosomal recessive phenotypes when only a heterozygous variant is found; generally the chance of there being a second variant is greater than the chance the person is an incidental carrier.

As the volume of genetic data generated per person continues to increase (from exon-by-exon analysis, through gene panels, to exomes, and now whole genome sequencing), this has inevitably led to a greater reliance on variant detection programs. The limitations of these algorithms to detect splicing variants, especially those occurring in U12 introns and less well defined consensus sequences, needs to be better recognized and urgently remedied (for instance, by the use of the Spliceman program, see Table. 1), otherwise, the full potential of genetic testing will be limited [10]. Until then, researchers in clinical laboratories should be vigilant in seeking splicing variants and perhaps should hand-curate for rare variations occurring beyond −1, −2, +1, +2 sites. If splicing variants that affect function are missed by splicing prediction programs, or by a conservatism to prevent the identification of too many variants of unclear significance in clinical laboratories, then this has two important consequences. Firstly, it decreases the utility of exome/genome scale sequencing, and secondly, it increases the risk that other variations may be erroneously regarded as disease-causing.

## Electronic supplementary material

Supplementary Materials
