# Supplementary material for: Before progressing from “exomes” to “genomes”… don’t forget splicing variants
Source: Eur J Hum Genet. 2018 Jul 12;26(11):1559–62. doi: 10.1038/s41431-018-0214-3 (PMC6189206; doi:10.1038/s41431-018-0214-3)
Supplement: Supplementary file 1 — Supplementary Materials [file 41431_2018_214_MOESM1_ESM.docx]

**Shaikh S et al. Viewpoint: Before progressing from “exomes" to “genomes”… don't forget splicing variants**

**Supplementary data: Methods**

The minigene vector (provided by Dr Allan Richards) contains exons 43-46 and introns 43-45 of *COL2A1*. Minigene constructs were made by the introduction of the test region between exon 44 and 45. For the *NTRK1* constructs, exon 6 and flanking introns were amplified using control and patient gDNA as the template. For the SCN9A constructs, exon 3, intron 3, and exon 4, with adjacent 200bp of the flanking introns were amplified from control and patient gDNA. This approach was used as the intron between exon 3 and exon 4 is a U12 intron, but the other two introns are canonical U2. The minigene assay was performed using HeLa cells, which were cultured in complete DMEM supplemented with 10% FBS, 2 mM l-glutamine and 100 μg/ml penicillin and 100 μg/ml streptomycin, at 37°C and 5% CO2. Cells were plated at a seeding density of 1.25 x 10^5^/ml and were transiently transfected with 2.5 μg of DNA using Fugene HD (Promega). Twenty-four hours post transfection, total RNA was extracted from cells using Qiagen RNeasy mini kit. One μg of total RNA was converted into cDNA using SuperScript III First-Strand Synthesis kit (Thermofisher) according to manufactures’ protocol. Instead of random oligonucleotides, a gene specific primer downstream of *COL2A1* exon 46 was used. The cDNA was then amplified using exon spanning primers: forward primer spanned exon 43 and exon 44 and the reverse primer spanned exon 45 and 46. Products were then separated by gel electrophoresis, bands excised and purified DNA was sequenced using the amplification step primers.
